# Walking for Transport among Older Adults: A Cross-Sectional Study on the Role of the Built Environment in Less Densely Populated Areas in Northern Germany

**DOI:** 10.3390/ijerph17249479

**Published:** 2020-12-17

**Authors:** Tanja Brüchert, Pia Hasselder, Paula Quentin, Gabriele Bolte

**Affiliations:** 1 Institute of Public Health and Nursing Research, Department of Social Epidemiology, University of Bremen, Grazer Str. 4, 28359 Bremen, Germany; t.bruechert@uni-bremen.de (T.B.); pi_ha@uni-bremen.de (P.H.); 2 Health Sciences Bremen, University of Bremen, 28359 Bremen, Germany; 3 Department of European Planning Cultures, Faculty of Spatial Planning, TU Dortmund University, August-Schmidt-Straße 10, 44227 Dortmund, Germany; paula.quentin@tu-dortmund.de

**Keywords:** built environment, age-friendly environment, neighborhood, active mobility, walking for transport, older adults, active aging

## Abstract

In the last decades, there has been rising interest in public health research in the importance of the built environment for a healthy and active life in old age, but little attention has been paid to less densely populated areas. This study aimed to explore the impact of the built environment on walking for transport in the context of an older population living in communities of <100,000 inhabitants. Within the project *AFOOT–Securing urban mobility of an aging population*, a cross-sectional postal survey was carried out from May to September 2019 in older adults (≥65 years) in the Metropolitan Region Northwest, Germany. Self-reported data from 2189 study participants were analyzed. Logistic and linear regression models were used to examine the associations between the built environment and walking for transport. Any walking and frequent walking were positively associated with nearly all built environment attributes, even after adjustment for demographic and health covariates. The amount of walking in minutes per week was associated only with residential density. Moderating effects of gender, age, and use of walking aids were identified. Improving the built environment appears to be a promising opportunity to motivate and enable older adults to walk for transport.

## 1. Introduction

There is an increasing trend to create livable and healthy cities focusing on more sustainable and people-oriented concepts (e.g., by banning private cars from the city center and make walking and cycling more attractive) [1,2]. However, up to now, far too little attention has been paid to less densely populated areas.

In Germany, almost two thirds of the population live in small and medium-sized towns [3]. This is especially true for older adults. Due to the migration of young people to the cities and increasing life expectancy, the proportion of older people in small and medium-sized towns, suburbs, and rural areas is growing [4]. In these less densely populated areas, poor public transport and longer distances usually go along with lower prevalence of active transport modes and even higher car use than in larger cities [5].

Overall, walking is the second most common transport mode among older adults [5] and according to the World Health Organization (WHO) recommendations on physical activity for health, walking is considered a moderately intensive activity [6]. Considering the low levels of physical activity in older adults in Germany, walking for transport (i.e., to the supermarket and other daily activities) is a promising opportunity to integrate an adequate level of physical activity in the everyday life of older adults. Physical activity is key to the maintenance of mobility and healthy aging. Physical activity has been found to reduce the risk of cardiorespiratory diseases, some cancers and dementia, and to increase well-being, cognitive function, and life-expectancy [7,8]. Furthermore, walking as a mode of transport enables participation in daily activities. Being able to go out and about in the neighborhood in old age enables people to be independent and maintain social contacts, which is crucial for healthy aging [9]. A recent study of older adults in metropolitan regions in Sweden demonstrated the importance to have the choice of different mobility options, especially public transport, to be able to participate in daily activities [10].

According to the ecological model of active living, the built environment has a major impact on the choice for active modes of transport [11]. In the last decades, public health research has been guided by this model to explore the influence of the built environment on a healthy and active life in old age [12,13]. In a recent review and meta-analysis of quantitative studies, the proximity and diversity of destinations, street connectivity, population density, proximity to public transport and parks as well as pedestrian friendly features were identified as consistent determinants of walking for transport among older adults [12]. It has been shown that the close proximity to destinations especially determines the choice to walk for transport in different countries across the world. Population density mainly serves as a proxy of the availability of destinations and street connectivity as a facilitator to access these destinations [12]. However, very little was found in the literature on the question whether this relationship is consistent in less densely populated areas. Most studies have been conducted in North America or dense urban areas in Asia, which limits transferability of different mobility cultures, infrastructures, and urban design to less densely populated areas in Europe [12,14,15]. In rural communities or small towns, the number of accessible destinations might be lower and the average distance to these destinations longer. Moreover, further studies have highlighted the impact of aesthetically pleasing environments on walking for transport among adults across 12 countries worldwide [16] and among older adults from Asia [17,18,19]. Qualitative studies stress the importance of traffic safety for walking [12,20]. However, the before-mentioned meta-analysis resulted in a nil association regarding both aesthetics and traffic safety [12]. As these aspects are highly influenced by perception, they might show cultural differences and therefore need further investigation. Only nine out of 42 studies included in the review and meta-analysis were from Europe and more than three-quarters had sample sizes below 1000 participants [12].

Furthermore, very little attention has been paid in previous studies to the moderating role of gender, age, and physical impairments on the association between the built environment and walking for transport among older adults [12]. Overall, women have a higher life expectancy and are at higher risk of old-age-poverty [21]. Across the life course, more women than men walk for errands [22], which may be due to the fact that they often have less access to a car [23]. Therefore, they are more dependent on alternative mobility modes such as walking. In addition, physical impairments increase with age and environmental barriers may limit the mobility of people because of fear of falling [24,25]. These gender, age, and physical impairment aspects can affect the relationship between the living environment and walking. To the best of the authors’ knowledge, the role of using a walking aid as a moderator has not yet been examined.

This study aimed to contribute to the growing area of research by exploring the role of the built environment on walking for transport in the context of an older population living in communities of <100,000 inhabitants. The primary research question is: Which environmental attributes are associated with any walking for transport, frequent walking for transport, and the amount of walking for transport? A second question addresses the moderating effects of age, gender, and using a walking aid on the environment-walking for transport association. The findings should make an important contribution to plan for more healthy, active, and age-friendly environments in the future.

## 2. Materials and Methods

### 2.1. Study Design

A cross-sectional postal survey was carried out from May to September 2019 among people aged 65 years and older. In Germany, this population group is considered as older adults by the Federal Statistical Office, which improves comparability with national data. The study was conducted as part of the project *AFOOT—Securing urban mobility of an aging population* focusing on the mobility behavior and built environment of older adults [26]. Ethical approval was obtained from the University of Bremen ethical committee (ethics vote 20181205).

### 2.2. Data Collection and Sample

A self-administered questionnaire was sent to 11,000 adults aged 65 years and older living in eleven rural districts and two urban municipalities (<100,000 inhabitants) in the Metropolitan Region Northwest in Germany. The sample was randomly selected among the respective age-group by the respective 117 residents’ registration offices. All registration offices followed the same sampling strategy (i.e., the number of adults drawn per spatial unit was determined according to the proportion of older adults (≥65 years) in the respective district or municipality). The study team prepared the study documents (self-administered questionnaire, explanation letter about the aims of the study and data processing procedure, a document for the declaration of consent and a prepaid envelope) and organized the mailing process. Participants were asked to send back the filled questionnaire and the declaration of consent in the prepaid envelope. There was neither a reminder nor incentive to participate in the study. Finally, 2242 older adults were included in the study (total response rate = 20.6%). Participants not living independently (i.e., participants living in a retirement home (*n* = 49), and participants with missing data on all outcome variables (*n* = 4)) were excluded from this analysis. In total, 2189 respondents were eligible for this analysis.

### 2.3. Measures

The questionnaire was largely based on validated questions from national or international studies as detailed below. Focus group discussions with older adults (≥65 years) were conducted to pre-test the questionnaire before the start of the survey.

#### 2.3.1. Perceived Neighborhood Environmental Attributes

Referring to the widely used Neighborhood Environment Walkability Scale (NEWS) [27,28], participants were asked to answer several questions about their neighborhood environment. Most questions were carried over from the original instrument, and some questions were modified to better fit the German context.

To assess the *residential density*, participants were asked to estimate the frequency of different types of residences in their immediate neighborhood, from detached single-family residences to apartments or condos with more than 13 stories. The response categories were ‘All’, ‘Most’, ‘Some’, ‘A few’, and ‘None’. The items were weighted according to the NEWS scoring protocol and the subscale score was calculated as the sum of the weighted values.

Land use mix was assessed by asking participants about the walking distance from their home to nearby facilities and services. Various types of facilities and services were listed (small grocery store/supermarket, drugstore, bakery, café, restaurant, physician, pharmacy, post office, bank/credit union, salon/barber shop, cemetery, recreation center) and four response categories were provided: ‘1–10 min’, ‘11–20 min’, ‘21–30 min’, and ‘31+ min’. The response categories were scaled from 4 (‘1–10 min’) to 1 (‘31+ min’), higher values indicating a closer proximity. The mean across all items was calculated to assess the *proximity of destinations. Destinations within 20-min walk* was calculated by the number of facilities and services within 20-min walking distance. The *proximity of bus stop* was assessed by asking participants about the walking distance to the nearest bus stop. Information on the proximity of the bus stop were separately included in the analysis because former research identified it as a possibly important factor to engage in active mobility [12].

To assess *walking infrastructure, cycling infrastructure, shared infrastructure* (for walking and cycling), *street connectivity, aesthetics and traffic safety,* participants were asked ‘What does the environment/streetscape in your neighborhood predominantly look like?’. They rated statements on a 4-point scale from ‘Strongly agree’ to ‘Strongly disagree’. For all these neighborhood environmental attributes, subscale scores were calculated as the mean of all items per subscale. Further details on the exact wording of the statements can be obtained from the appendix (Appendix A).

#### 2.3.2. Walking for Transport

In this study, we assessed walking as a mode of transport (e.g., to go shopping, to commute to work or to visit friends or family). Walking for transport was explored by three different outcomes: any walking for transport, frequency of walking for transport, and amount of walking for transport.

Participants were asked ‘Do you walk or cycle for transport with a duration of at least 5 min?’ and could answer with ‘Yes, I walk for transport’ and/or ‘Yes, I cycle for transport (using a regular bike)’ and/or ‘Yes, I cycle for transport (using an electric bicycle/pedelec)’ or ‘No’. *Any walking for transport* was used as a dichotomous outcome: ‘Yes, I walk for transport’ was considered a ‘Yes’, not choosing this option was considered a ‘No’.

*Frequency of walking for transport* was assessed by asking participants how often they usually walk for transport. Response options were: ‘Daily or almost daily’, ‘3–4 days a week’, ‘1–2 days a week’, ‘1–2 days a week’, ‘1–3 days per month’, ‘Less often’, or ‘Never’. According to their answer, respondents were either classified into the category ‘≥3 days a week’ or ‘<3 days a week’.

*Amount of walking for transport* was measured in minutes per week. The questions used to assess this aspect of active mobility were based on similar questions from the International Physical Activity Questionnaire long-form (IPAQ-LF). They were modified to better meet the specific needs of older adults (≥65 years) as the original questions were created for adults from 15 to 69 years of age. It has been shown that older adults may benefit from even small amounts of physical activity [7], or by just leaving the house [9]. Accordingly, participants were asked about activities with a duration of at least 5 min in this survey instead of 10 min as in the original instrument. Respondents were asked to specify the number of days per week they typically engage in walking for transport. Additionally, they were asked to estimate the time spent for this activity in hours and minutes per day. Based on the information provided in response to these two questions, the sum of minutes per week was calculated. A value of 1260 min per week (corresponds to 180 min per day) was defined as the maximum time per week. Higher values were recoded to this maximum value following the scoring guidelines from IPAQ [29].

#### 2.3.3. Socioeconomic Variables

*Gender* was assessed as a dichotomous variable (‘Male’, ‘Female’). Information about the *age* of the participants was gathered by asking participants to fill in their year of birth. *Age groups* were built with respect to quartiles (’65–69 years’, ’70–74 years’, ’75–79 years’, ’80 years and over’).

Information about the relationship status were summarized as *partner status* with the categories ‘Partner’ and ‘No partner’. *Living situation* was categorized in ‘Living alone’ and ‘Not living alone’. Concerning the *area of residence*, participants were asked to name their municipality. Based on the classification of the Federal Institute for Research on Building, Urban Affairs and Spatial Development [30], the named municipalities were categorized into four groups: ‘Medium-sized town’ (20,000–99,999 inhabitants), ‘Larger small town’ (10,000–19,999 inhabitants), ‘Small town’ (5000–9,999 inhabitants or at least basic central function), and ‘Rural community’ (<5000 inhabitants).

Information about school education and professional education were summarized as *education* according to the International Standard Classification of Education (ISCED) [31] and classified into three categories: ‘Low’ (ISCED 0–2), ‘Middle’ (ISCED 3–4), and ‘High’ (ISCED 5–6).

*Income* was assessed by asking participants to give information about their monthly net income and their household composition (number and age of household members). Following the guidelines of the Organisation for Economic Co-operation and Development (OECD) [32], the equivalized disposable income was calculated. With respect to the latest poverty line of Lower Saxony [33], three categories were used to classify the level of income: ‘Low’ (<60% median), ‘Middle’ (≥60% median and ≤median), and ‘High’ (>median).

*Country of birth* was assessed by asking participants to mark either ‘In Germany’ or ‘In another country’ in response to the question ‘In which country were you born?’. Additionally, participants were asked to specify the country of birth in a free text field if other than Germany. Country of birth was analyzed as a dichotomous variable (‘Germany’, ‘Other country’).

#### 2.3.4. Health

*Self-rated health* was measured on a 5-point scale ranging from ‘Very good’ to ‘Very poor’ [34]. Respondents were grouped into the categories ‘Very good, good’ and ‘Moderate, poor, very poor’.

Participants were also asked about *mobility impairments*. Different mobility impairments were listed, and multiple responses were possible. The given information was categorized into two groups: ‘At least one’ and ‘None’.

The use of a *walking aid* was explored by asking participants ‘Do you use a walking aid?’. A ‘Yes’ could be specified as ‘walking stick/cane’ and/or ‘crutches’ and/or ‘walker’ and/or ‘walking frame’ and/or ‘quad walker’ and/or ‘wheelchair’. A dichotomous variable (‘Yes’/’No’) was computed for this analysis.

#### 2.3.5. Transport

*Driving license* was assessed by asking participants whether they have a driving license or not (‘Yes’/’No’).

The question ‘How often can you use a car as a driver or passenger?’ was used to gather information about *car availability*. The response options were ‘Always’, ‘Sometimes’, and ‘Never’.

*Bicycle availability* (‘Yes’/’No’) was derived from the reported ownership of a regular bicycle and/or an electric bicycle/a pedelec and/or a tricycle. The availability of at least one of the above-mentioned vehicles was considered as ‘Yes’.

### 2.4. Analyses

All analyses were performed with SAS 9.4 Software (SAS Institute, Cary, North Carolina, US).

Univariate descriptive analyses were performed for all variables related to the research question of this study. Absolute and relative frequencies for a range of variables describing sociodemographic, health, and mobility characteristics of the study participants were calculated and stratified by gender. Bivariate statistical analyses of the study population characteristics with the three outcome variables were computed using chi-square tests for dichotomous outcome variables and Wilcoxon or Kruskal–Wallis tests for discrete outcome variables.

The associations between perceived neighborhood environmental attributes and any walking for transport and the frequency of walking for transport were analyzed via logistic regression models. Results are presented as odds ratios (OR) with corresponding 95% confidence intervals (CI). All environmental variables were treated as continuous variables and recoded so higher scores indicated a more favorable value of the environmental variable [27].

Linear regression models were used to examine associations of environmental attributes with amount of walking for transport in minutes/week. A log-linear model was fitted to these variables as the empirical distributions in min/week were positively skewed. To account for overdispersion, a negative binomial regression with canonical log link function was performed. In comparison to Poisson and log-normal regression, this model showed the best fit in terms of Akaike Information Criterion (AIC), deviance, and Pearson chi-squared statistics. Results are presented as the exponential of L’Beta with corresponding 95% confidence intervals. It can be interpreted as the proportional increase or decrease in minutes per week walking associated with changes in the environmental attributes.

Crude and adjusted models were run for each environmental attribute and outcome separately. The adjusted models additionally included gender, age, partner status, area of residence, education (ISCED), income, use of walking aid, car availability, and bicycle availability as covariates. These covariates were chosen as they were positively associated with one or more outcome variables in the previous tests of association (*p*-value < 0.05). The covariate of car ownership was recoded for adjusted models with the categories ‘Sometimes’ and ‘Never’ collapsed into one category because five participants at most fell in the category ‘Never’.

Separate models were run to estimate the moderating effects of gender, age, and use of a walking aid on the environment-walking for transport association. As this approach is exploratory, a probability level of 0.1 was set to indicate the possible interaction effects according to Hosmer et al. [35]. All environmental attributes with an interaction effect significant at a 0.1 probability level with any active mobility outcome in crude models are presented as stratified results. Adjusted models were run with the same covariates as in the main analysis.

## 3. Results

### 3.1. Characteristics of the Study Population

Table 1 presents the characteristics of the study population overall and stratified by gender.

Overall, the participants were 65 to 99 years old with a median age of 73 (data not shown). More than half of the participants were men (53%). More men than women had a partner (86% vs. 69%) and lived together with at least one other person (85% vs. 73%). The majority of the study participants lived in small and medium sized towns, only 8% of the total sample lived in a rural community. A total of 97% of men and 87% of women had at least a middle level of education according to ISCED, while the prevalence of a high level of education was two times greater in men than in women (47% vs. 23%). Overall, 14% of the participants had an equivalized disposable income below the poverty line of the respective region (Lower Saxony). Nearly the whole sample was born in Germany (97%).

Concerning health, almost 60% of the study participants self-rated their health as good or very good. More than a third of the total sample reported a mobility impairment and 14% the use of a walking aid. There were no great differences between women and men.

In men, nearly all participants had a driving license (97%) and always had access to a car (94%) whereas in women, these proportions were considerably smaller (89% and 84%). In our study population, there was also a high prevalence of bike ownership, but again, the availability of a bicycle was less common among women than men (80% vs. 88%).

### 3.2. Prevalence of Walking for Transport

Table 2 shows the participants’ engagement in walking for transport separately for all three outcomes by sociodemographic characteristics, health status, and transport aspects.

More than half of the participants walked at least three days a week. Seventy-one percent walked for transport at least 5 min, among these study participants, the median minutes walked per week were 120 min. The frequency of walking for transport differed by gender with the result that men tended to engage more often in walking for transport than women. Furthermore, any walking and the frequency of walking for transport differed by age groups. First, prevalence increased from 65–74 years. From the ages of 75 years onward, the engagement in walking for transport decreased with respect to these two outcomes. There were no significant differences in partner status, living situation, and area of residence for the investigated outcomes of walking for transport. However, there was a tendency of higher prevalence of engaging in any walking in areas of residence with a larger population.

Engagement in any walking and frequent walking for transport increased with higher education. Furthermore, a higher income was positively associated with frequent walking for transport, while it was, on the other hand, negatively associated with the amount of walking for transport. People with good health conditions (very good/good health, no mobility impairments, no walking aid) were more likely to walk at all and to walk frequently for transport. Concerning transport aspects, having a driving license was not associated with any of the outcomes. However, having no access to a car or bicycle was associated with more weekly minutes of walking for transport. Having a bicycle was furthermore more prevalent among people who engaged in any walking and frequent walking for transport.

### 3.3. Associations of the Built Environment and Walking for Transport

Table 3 summarizes the results of the logistic and linear regression analyses.

The denser the area of residence, the more likely the participants were to walk at all and the more frequently they walked. Each unit increase in residential density was associated with 1% higher odds (95% CI = 1–1%) of engagement in any walking and frequent walking for transport and <0.1% for amount of walking.

Walking infrastructure was positively associated with any walking and frequent walking for transport. Each unit increase in walking infrastructure was associated with 36% higher odds of engagement in any walking (95% CI = 21–53%) and 33% higher odds of engagement in frequent walking for transport (95% CI = 19–49%). In contrast to walking infrastructure, cycling infrastructure was not associated with any of the three outcomes. However, the perception of having shared infrastructure in the environment was associated with 13% higher odds of any walking for transport (95% CI = 3–24%).

Street connectivity showed a positive association with increased odds of over 67% of walking at all (95% CI = 44–95%) and 64% of walking frequently (95% CI = 42–89%) the better the street connectivity.

Concerning aesthetics, the results also showed positive associations with any walking for transport [OR = 1.30 (95% CI = 1.13–1.50)] and with the frequency of walking for transport [OR = 1.25 (95% CI = 1.09–1.43)]. Comparable associations were observed for traffic safety. Each unit increase in traffic safety was associated with 22% higher odds of engagement in any walking for transport (95% CI = 4–43%) and 16% higher odds of engagement in frequent walking for transport (95% CI = 0.3–35%).

Proximity of destinations was the environmental attribute showing the strongest association across all investigated environmental attributes. With each increase in proximity of several destinations by 10 min, the odds of engaging in any walking for transport at all [OR = 1.82 (95% CI = 1.61–2.06)] or walking for transport at least three days a week [OR = 1.88 (95% CI = 1.67–2.11)] nearly doubled. An increasing number of destinations within a 20-min walk also showed positive associations with any walking [OR = 1.12 (95% CI = 1.09–1.15)] and frequent walking [OR = 1.12 (95% CI = 1.09–1.15)]. Comparably, the closer the bus stop (proximity of a bus stop), the higher the odds of engaging in any walking for transport [OR = 1.19 (95% CI=1.08–1.32)]. Even higher were the odds of frequent walking for transport [OR = 1.33 (95% CI = 1.21–1.47)] with increasing proximity of a bus stop.

### 3.4. Moderating Effects

Table 4 presents the moderating effects of gender, age, and use of a walking aid on the environment-walking for transport association.

The positive association between street connectivity and any walking for transport decreased with age. Only among the younger age groups (≤69 years and ≤74 years, respectively) did positive associations with aspects related to cycling infrastructure and aesthetics appear. Among the oldest age group (80+ years), minutes walked per week decreased despite an increase in the perception of traffic safety.

The positive association of infrastructure features (walking, cycling, and shared infrastructure) and aesthetically pleasing environments with frequent walking was moderated by use of a walking aid. For example, for people with a walking aid, the chance to walk frequently was nearly twice as high with better walking infrastructure and aesthetical pleasing environments.

Proximity to destinations was found to be more strongly positively associated with the amount of walking for transport in women than men.

## 4. Discussion

The first research question in this study addressed the relationship between the built environment and different outcomes of walking for transport among older adults living in less densely populated areas in Germany. It was found that any walking and frequent walking were positively associated with nearly all built environment attributes. Modeling with different demographic and health covariates demonstrated the robustness of the findings that the higher or better the density, street connectivity, walking infrastructure, aesthetics, traffic safety, proximity of destinations, number of destinations within 20-min walk, and proximity of a bus stop, the higher the chance to walk for transport and to do it at least three days a week. Any walking was additionally related to shared infrastructure. The amount of walking in min per week was associated only with residential density.

In terms of the second research question, moderating effects were found especially for age and the use of a walking aid.

### 4.1. Residential Density

This finding broadly supports the work of other studies in this area, linking higher residential density with a higher frequency of walking for transport among older adults [17,18,19]. Although these studies were all conducted in very dense urban environments in Asia, we can also confirm these findings for less densely populated areas in Germany. However, the odds were quite small, reflecting the results of Kerr et al. [16] who compared adults of 17 cities in 12 countries. The positive effect of residential density on the probability of walking may only occur above a certain density level, so the effects in our study remained rather small. This is not surprising as higher density often just indicates shorter distances to destinations, which is an important determinant of walking behavior among older adults, as discussed in the next section.

### 4.2. Proximity to Destinations

The most important built environment attribute for any walking and frequent walking in our study was proximity to destinations as a measure of land use mix. This is in line with other studies that used comparable methods [17,36,37]. Cerin et al. [12] even concluded in their meta-analysis that the proximity to destinations was the driving force of active mobility in older adults. Our study confirmed these results for less densely populated areas in Germany. The evidence based on the relationship of proximity to destinations and number of reachable destinations with the amount of walking for transport in minutes walked per week was less consistent. Although most studies showed a positive relationship [17,38,39], King et al. [40] found no relationship comparing five sites across the U.S. In our study, proximity to destinations was also not associated with more minutes of walking for transport per week in the whole study population after adjusting for several covariates. However, taking possible effect modification into account, it was positively associated among women, but not among men, which is in line with findings from other studies [15,41]. The findings may be due to gender roles, for example, more women than men are responsible for shopping [42]. Furthermore, since women of this generation are less likely than men to have access to a car [43], they are dependent on other forms of mobility such as walking. Accordingly, it is important to preserve close access to destinations and service providers even in less densely populated areas in order not to disadvantage women. Overall, the proximity of destinations was associated with any walking and frequent walking. If people have the possibility to walk to destinations nearby and do it more than once a week, they move their body on a regular basis. They also have social contacts while walking and doing the shopping, preventing social isolation. Social isolation increases the risk of a variety of physical and mental health conditions such as heart disease and cognitive decline and is therefore an important public health concern [44,45].

The positive association between proximity to public transit and walking for transport is consistent with former studies [37,46]. However, in some places of the area under study, public transport services are rare. Rail transit may not be accessible and buses might run only a few times a day, which makes public transit unattractive. Further research is needed that includes information on the frequency of bus service and running hours.

### 4.3. Street Connectivity

Street connectivity was the second most important determinant of any and frequent walking, which confirms findings from former studies [17,39,47]. In accordance with other studies, street connectivity has furthermore been shown not to be related to minutes walked per week in older adults [40]. High street connectivity is known as proxy for short distances and the possibility of taking various routes to a destination. Our study results support the importance of this environmental attribute for walking in old age, even in less densely populated areas.

### 4.4. Infrastructure

The positive association of walking infrastructure with walking for transport is in line with other studies [17,37], while results on shared infrastructure have been mixed in previous studies [18,19]. In our study, walking infrastructure was the third most important attribute for any walking and, together with proximity of a bus stop, for frequent walking. In the region under study, footpaths are frequently also used by cyclists, especially in rural areas. The fact that shared infrastructure improved the odds of any walking implies that any infrastructure separating pedestrians from motorized traffic can foster walking, even if cycling is also allowed. However, qualitative studies have shown that cycling on the footpaths disturbs pedestrians [20]. In accordance with these studies, the odds for any walking were higher for walking infrastructure compared to shared infrastructure. Walking, cycling, and shared infrastructure were furthermore found to be more strongly positively associated with frequent walking in people using a walking aid. Considering the importance of preventing falls in older adults [25], this important finding has to be taken into account in future planning.

### 4.5. Aesthetics

Although positive relationships between walking and aesthetically pleasing environments have been found in Asian contexts [17,18,19,36], studies from Belgium showed no association [37,39]. The meta-analysis by Cerin et al. [12] supports the thesis of a nil association. A study from Denmark examining the relationship between aesthetics and the amount of walking for transport, furthermore found no differences between frail and non-frail older adults [38]. Despite these studies, we found evidence for the positive association of aesthetics and walking for transport, especially for people using a walking aid and for people of younger age (i.e., under 74). We assume that the perception of aesthetically pleasing environmental attributes is related to cultural, socio-economic, and generational differences. The results on aesthetics should therefore always be interpreted with caution considering the context of the study and additional individual factors of the participants.

### 4.6. Traffic Safety

Observational studies have consistently shown that there is no association between traffic safety and walking for transport among older adults [12]. Qualitative studies, in contrast, point to the importance of safety issues for older adults [20]. The latter is supported by our results as we found a positive association of traffic safety with any walking and frequent walking for transport. Nevertheless, our results have also shown that even with rising traffic safety, the number of minutes walked per week decreases among the oldest age group (above 80). The discrepancy could be attributed to the role of perception. Studies from Germany have shown that older adults tend to perceive more danger from traffic and crime than objective measures indicate [48,49]. However, safety concerns that discourage walking are not groundless and should not be ignored. An older person falling out of fright or when trying to avoid a car might not even be registered as an accident in official statistics. Falls may, however, be a huge disruption in that person’s life and even trigger a need for care [25,50].

### 4.7. Strengths and Limitations

This is one of the first studies in Germany describing the association of the built environment on walking for transport among older adults. As former national and international studies have been conducted mostly in urban areas [12], our focus on communities with <100,000 inhabitants sheds light on the impact in less densely populated areas. Dependence on driving and limited public transport services characterize these areas. It was found that almost all study participants had access to a car, so walking was an optional activity. We assume that environmental conditions show stronger effects when walking is not the only available means of transport. This strengthens our conclusions on the relationship between the environment and walking as a means of transport. Another strength of this study is that cycling infrastructure, pedestrian infrastructure, and shared infrastructure have been assessed separately, providing differentiated information for urban and transport planning.

In observational studies, there is a potential for bias from self-selection. In terms of generalizability, the results therefore need to be interpreted with caution. The high proportions of men, married people, and people with good or very good health condition in our study did not correspond with those evident in the general population [51,52,53]. Willingness to participate may have resulted in a study population, which is more active than the general population of that age group. However, the proportion of persons regarding education, household income, country of birth, and residential area are comparable with the general population of Lower Saxony and can therefore be interpreted as almost representative for the population [33,54,55,56].

Another source of uncertainty is that older adults tend to overreport their physical activity using the IPAQ questionnaire [57]. In addition, the results for the amount of walking in our study indicate that study participants did not exactly calculate minutes walked per week, but did estimate an average value. This is expressed by the fact that the median of the minutes walked per week was 120 min on average (i.e., 2 h/week) and, therefore, no major differences could be observed between groups (see Table 2). Furthermore, although the questionnaire provided separate questions on walking for transport and walking for recreation, it might be possible that the respondents did not fully differentiate between walking for transport and other walking activities. It is important to bear in mind the possible bias in these responses, although we assume that this potential misclassification is independent of the built environment.

A last note of caution is due here since this is a cross-sectional study and, therefore, no conclusion can be drawn on the causal pathway between the built environment attributes and walking for transport.

### 4.8. Implications

Cities and municipalities could make an important contribution with better framework conditions for active mobility and thus motivate the population to walk more. Intersectoral action on the administrational level between planners and health professionals is crucial to adequately address the needs of older adults. By including health needs and impacts in the conception, design, and planning of urban design measures, policy makers and urban and transport planners can stimulate the development of sustainable communities [2].

The positive association between higher residential density and more walking for transport supports the focus on inner-city development instead of urban sprawl through new building areas outside the centers. When new development areas are designated, care should be taken to allow multiple access routes for pedestrians and cyclists and to ensure the proximity and accessibility of services (e.g., shops and educational facilities). This also applies to conversion measures in existing areas. In addition, new development areas may encourage walking when they are closed for car traffic. Implementing such a strategy would promote safety in the community for children as well as for older people.

When rebuilding roads, the distribution of space should be carefully reconsidered, and the needs of vulnerable groups taken into account. Paths for pedestrians only should be preferred over shared solutions to foster walking for transport. Both moving and parked motorized traffic have a large amount of space at their disposal in cities and municipalities, which should urgently be questioned in terms of equal opportunities for all road users. Instead of stirring up competition between environmentally-friendly means of transport in shared spaces, space should be taken away from the space-taking motorized traffic, which can also improve feelings of safety [58].

Even if it is not possible to change the built environment overnight, development plans, conversion measures, or improvements in infrastructure are put forward and implemented continuously in the municipalities. Here, attention should be paid to the needs of the growing group of older adults in order to enable healthy aging. The public health service should get involved with other sectors to ensure healthy aging and its determinants are addressed. In Germany, such involvement of the public health services in planning processes is possible within the framework of the participation of public interest agencies. Furthermore, it can contribute information on the health effects of the built environment to local and regional committees or working groups dealing with health, aging, or the built environment. Together with evidence from other studies, our results should be integrated in public participation processes to explain the need for such changes and to gain the support of the population. However, the needs of the city or community as a whole must always be kept in mind to avoid environmental health inequalities [59], since people who participate in participation processes are often a selective group [60,61].

For future research, special attention should be paid to the attributes of the perception of safety and aesthetics. Cultural, generational, and biographical (e.g., history of falls) factors should be considered to better explain the relationship between perceived safety and aesthetics and walking for transport among older adults, also in comparison to other countries. This could be followed by developing evidence-based assessment tools that are better suited to assess the needs of an aging population and that inform urban and transport planners in municipalities about adaptations needed to be made in the environment. These should assess details on the street design such as surface quality, lighting, wayfinding, or crossings to help to reduce obstacles for walking [20,62,63]

## 5. Conclusions

Built environment attributes to foster walking for transport among older adults are also important in less densely populated areas. The proximity of destinations is especially crucial, but street connectivity and pedestrian infrastructure are also important. In the face of an aging population, these environmental attributes should be considered in urban planning to ensure independent living, especially in rural areas. Public health services have an important role to play in strongly advocating for the special needs of older people in planning processes to enable healthy aging through active mobility.

## Figures and Tables

**Table 1:** Characteristics of the study population.

	Total (n = 2189)		Male (n = 1115)		Female (n = 996)	
	**n**	**%**	**n**	**%**	**n**	**%**
**Age, years**						
65–69	593	28.1	303	27.2	290	29.1
70–74	538	25.5	275	24.7	262	26.3
75–79	508	24.1	273	24.5	234	23.5
80+	472	22.4	262	23.5	210	21.1
**Partner status**						
Partner	1643	77.9	954	85.7	687	69.2
No partner	465	22.1	159	14.3	306	30.8
**Living situation**						
Living alone	455	20.9	167	15.0	271	27.3
Not living alone	1727	79.2	946	85.0	723	72.7
**Area of residence**						
Medium sized town	653	30.2	320	29.1	316	32.1
Larger small town	795	36.8	392	35.6	367	37.3
Small town	549	25.4	301	27.3	229	23.3
Rural community	165	7.6	88	8.0	72	7.3
**Education, ISCED**						
Low	165	7.7	33	3.0	128	13.1
Middle	1229	57.1	547	49.8	631	64.3
High	760	35.3	518	47.2	222	22.6
**Income**						
Low	287	13.6	144	13.3	136	14.2
Middle	699	33.0	340	31.3	331	34.6
High	1130	53.4	601	55.4	490	51.2
**Country of birth**						
Germany	2029	96.5	1077	97.0	951	96.1
Other country	73	3.5	33	3.0	39	3.9
**Self-rated health**						
Very good/good	1272	58.8	642	58.3	576	58.4
Moderate/poor/very poor	893	41.3	460	41.7	410	41.6
**Mobility impairments**						
At least one	824	38.2	416	37.7	391	40.0
None	1334	61.8	689	62.4	586	60.0
**Walking aid**						
Yes	311	14.3	144	13.0	160	16.2
No	1862	85.7	966	87.0	825	83.8
**Driving license**						
Yes	1996	93.3	1065	97.3	860	88.7
No	143	6.7	30	2.7	110	11.3
**Car availability**						
Always	1910	89.3	1029	94.0	813	83.8
Sometimes	160	7.5	42	3.8	115	11.9
Never	70	3.3	24	2.2	42	4.3
**Bike availability**						
Yes	1839	84.6	978	88.2	786	79.7
No	334	15.4	131	11.8	200	20.3

**Table 2:** Walking for transport by sociodemographics, health status, and transport aspects.

	Any Walking for Transport			Frequency of Walking for Transport			Amount of Walking for Transport			
	Yes			≥3 Days a Week			Min/Week			
	n	%	*p*-Value ^a^	n	%	*p*-Value ^a^	n	Median	IQR	*p*-Value ^b^
**Gender**										
Male	785	71.4	0.7597	601	55.9	0.0361	744	120	180	0.6882
Female	699	70.8		478	51.2		641	120	165	
**Age, years**										
65–69	420	71.3	0.0035	316	55.5	0.0348	405	120	180	0.1701
70–74	396	74.3		287	56.2		373	120	160	
75–79	368	73.6		264	54.9		330	120	210	
80+	300	64.7		213	47.8		277	120	180	
**Partner status**										
Partner	1155	71.3	0.8431	837	53.5	0.7216	1081	120	180	0.2312
No partner	327	70.8		239	54.4		301	120	210	
**Living situation**										
Living alone	337	74.4	0.1474	235	54.4	0.8868	309	120	210	0.3152
Not living alone	1206	70.9		888	54.0		1131	120	180	
**Area of residence**										
Medium sized town	487	75.2	0.0512	357	56.8	0.4471	453	120	180	0.3302
Larger small town	560	71.4		400	53.3		529	120	165	
Small town	372	69.1		272	52.3		346	120	180	
Rural community	109	66.5		84	53.5		100	120	225	
**Education, ISCED**										
Low	101	62.0	0.0005	68	43.9	0.0004	96	90	155	0.0734
Middle	857	70.7		611	52.6		796	120	180	
High	570	76.0		436	59.4		534	120	165	
**Income**										
Low	193	68.2	0.2744	121	45.2	0.0033	178	120	255	0.0155
Middle	501	72.9		359	53.9		468	120	210	
High	813	72.7		615	56.6		765	120	150	
**Country of birth**										
Germany	1419	70.8	0.2748	1027	53.3	0.2851	1331	120	180	0.8705
Other country	56	76.7		43	59.7		45	120	150	
**Self-rated health**										
Very good/good	949	75.5	<0.0001	711	58.5	<0.0001	892	120	180	0.6735
Moderate/poor/very poor	579	66.0		402	47.6		535	120	180	
**Mobility impairments**										
At least one	519	63.9	<0.0001	353	45.8	<0.0001	478	120	180	0.3602
None	1008	76.6		761	59.2		949	120	180	
**Walking aid**										
Yes	166	54.4	<0.0001	109	37.7	<0.0001	148	120	180	0.8522
No	1368	74.4		1007	56.6		1285	120	180	
**Driving license**										
Yes	1407	71.5	0.6206	1026	53.9	0.7806	1320	120	180	0.2763
No	105	73.4		75	55.2		92	120	180	
**Car availability**										
Always	1361	72.1	0.3791	994	54.5	0.3882	1275	120	180	0.0112
Sometimes	109	69.0		76	50.7		101	140	240	
Never	44	65.7		31	47.7		39	180	280	
**Bike availability**										
Yes	1340	73.6	<0.0001	969	55.3	0.0100	1263	120	180	0.0385
No	195	60.0		151	47.5		172	135	240	

IQR = Interquartile range; ^a^ *p*-value of Chi ^2^ test; ^b^ *p*-value of Wilcoxon or Kruskal–Wallis test.

**Table 3:** Associations between perceived neighborhood environmental attributes and any walking for transport, frequency of walking for transport, and amount of walking for transport.

	Any Walking for Transport			Frequency of Walking for Transport(≥3 Days a Week)			Amount of Walking for Transport(Min/Week)		
	n	Crude OR (95% CI)	Adjusted OR (95% CI)	n	Crude OR (95% CI)	Adjusted OR (95% CI)	n	Crude exp(β) (95% CI)	Adjusted exp(β) (95% CI)
**Density**	1854	1.01 (1.01–1.01)	1.01 (1.01–1.01)	1790	1.01 (1.01–1.01)	1.01 (1.01–1.01)	1253	1.00 (1.00–1.00) ^†^	1.00 (1.00–1.00) ^†^
**Walking infrastructure**	1796	1.38 (1.23–1.54)	1.36 (1.21–1.53)	1745	1.34 (1.20–1.49)	1.33 (1.19–1.49)	1223	1.02 (0.96–1.09)	1.01 (0.94–1.08)
**Cycling infrastructure**	1739	1.09 (0.95–1.25)	1.06 (0.92–1.22)	1680	1.07 (0.94–1.21)	1.04 (0.92–1.19)	1169	1.00 (0.93–1.08)	1.00 (0.93–1.07)
**Shared infrastructure (for walking and cycling)**	1852	1.14 (1.04–1.25)	1.13 (1.03–1.24)	1791	1.08 (0.99–1.17)	1.06 (0.97–1.16)	1250	0.98 (0.94–1.03)	0.99 (0.95–1.05)
**Street connectivity**	1804	1.70 (1.47–1.97)	1.67 (1.44–1.95)	1752	1.61 (1.40–1.84)	1.64 (1.42–1.89)	1219	1.06 (0.98–1.14)	1.05 (0.97–1.13)
**Aesthetics**	1697	1.35 (1.17–1.55)	1.30 (1.13–1.50)	1649	1.28 (1.12–1.46)	1.25 (1.09–1.43)	1153	0.99 (0.92–1.07)	0.99 (0.92–1.07)
**Traffic safety**	1814	1.27 (1.09–1.48)	1.22 (1.04–1.43)	1756	1.22 (1.05–1.40)	1.16 (1.00–1.35) ^†^	1220	0.94 (0.86–1.02)	0.93 (0.86–1.02)
**Proximity of destinations**	1899	1.90 (1.68–2.13)	1.82 (1.61–2.06)	1832	1.94 (1.73–2.17)	1.88 (1.67–2.11)	1283	1.07 (1.00–1.13)	1.06 (0.99–1.13)
**Destinations within 20-min walk**	1899	1.13 (1.10–1.15)	1.12 (1.09–1.15)	1832	1.13 (1.10–1.15)	1.12 (1.09–1.15)	1283	1.01 (1.00–1.03) *	1.01 (1.00–1.02) *
**Proximity of bus stop**	1899	1.25 (1.13–1.37)	1.19 (1.08–1.32)	1832	1.38 (1.26–1.52)	1.33 (1.21–1.47)	1283	1.02 (0.97–1.08)	1.01 (0.95–1.07)

OR = odds ratio; exp (β) = exponentiated L’Beta; CI = confidence interval; separate models were computed for the different environmental attributes. Two models were conducted for each environmental attribute and outcome (crude and adjusted). The adjusted models additionally included gender, age, partner status, area of residence, education (ISCED), income, walking aid, car availability, and bicycle availability as independent variables. ^†^ *p* < 0.05 * *p* ≥ 0.05.

**Table 4:** Effect modification by gender, age, and use of a walking aid of the association of perceived environmental attributes with walking for transport.

	n	Crude OR (95% CI)	Adjusted OR (95% CI)
***Any Walking for Transport***			
Cycling infrastructure * age			
65–69	527	1.48 (1.14–1.92)	1.47 (1.12–1.93)
70–74	460	0.94 (0.72–1.24)	0.91 (0.69–1.21)
75–79	403	0.98 (0.74–1.31)	0.96 (0.71–1.29)
80+	349	0.96 (0.73–1.26)	0.92 (0.69–1.23)
Street connectivity * age			
65–69	545	2.14 (1.61–2.85)	2.32 (1.70–3.16)
70–74	467	1.74 (1.28–2.35)	1.77 (1.29–2.43)
75–79	427	1.44 (1.07–1.92)	1.41 (1.04–1.90)
80+	365	1.52 (1.13–2.05)	1.44 (1.05–1.98)
Aesthetics * age			
65–69	512	1.57 (1.22–2.01)	1.58 (1.21–2.05)
70–74	453	1.42 (1.07–1.90)	1.41 (1.04–1.90)
75–79	396	1.18 0.88–1.59)	1.14 (0.84–1.55)
80+	336	1.17 (0.88–1.56)	1.06 (0.78–1.45)
	**n**	**Crude OR (95% CI)**	**Adjusted OR (95% CI)**
*Frequent walking for transport*			
Walking infrastructure * walking aid			
Yes	222	1.81 (1.29–2.55)	1.92 (1.33–2.78)
No	1523	1.29 (1.15–1.45)	1.29 (1.14–1.45)
Cycling infrastructure * walking aid			
Yes	206	1.43 (1.00–2.05)	1.40 (0.95–2.07)
No	1474	1.01 (0.88–1.15)	1.00 (0.87–1.14)
Shared infrastructure * walking aid			
Yes	233	1.33 (1.05–1.70)	1.44 (1.11–1.88)
No	1558	1.03 (0.93–1.13)	1.02 (0.93–1.13)
Aesthetics * walking aid			
Yes	203	1.77 (1.20–2.60)	1.87 (1.23–2.85)
No	1446	1.21 (1.06–1.39)	1.20 (1.04–1.38)
	**n**	**Crude exp(β) (95% CI)**	**Adjusted exp(β) (95% CI)**
*Walking amount min/week*			
Land use mix proximity * gender			
Male	699	1.02 (0.93–1.11)	1.01 (0.93–1.10)
Female	584	1.13 (1.03–1.23)	1.12 (1.02–1.22)
Traffic safety * age			
65–69	373	1.04 (0.90–1.21)	1.07 (0.92–1.24)
70–74	332	0.92 (0.78–1.09)	0.94 (0.79–1.12)
75–79	281	0.92 (0.77–1.10)	0.92 (0.77–1.10)
80+	234	0.80 (0.67–0.97)	0.82 (0.67–0.99)

OR = odds ratio; exp(β) = exponentiated L’Beta; CI = confidence interval. Separate models were computed for the different environmental attributes. Two models were conducted for each environmental attribute and outcome (crude and adjusted). The adjusted models additionally included age (if not a moderator), gender (if not a moderator), walking aid (if not a moderator), education (ISCED), income, living with a partner, area of residence, car ownership, and bike ownership.

## Appendix A

Statements on the assessed environmental attributes (based on the NEWS questionnaire).

*Walking infrastructure*

‘There are sidewalks on most of the streets in my neighborhood’
‘The sidewalks are well maintained (paved, even, and few potholes)’
‘The sidewalks are wide enough’

*Cycling infrastructure*

‘There are cycle paths on most of the streets in my neighborhood’
‘The cycle paths are well maintained (paved, even, and few potholes)’
‘The cycle paths are wide enough’

*Shared infrastructure*

‘There are shared paths for walking and cycling’

*Street connectivity*

‘There are many four-way intersections’
‘The distance between intersections is usually short (100 meters or less; the length of a soccer field or less)’
‘There are many alternative routes for getting from place to place (I don’t have to go the same way every time)’

*Aesthetics*

‘There are trees along the streets in my neighborhood’
‘Trees give shade for the sidewalks in my neighborhood’
‘There are many interesting things to look at while walking in my neighborhood’
‘My neighborhood is generally free from litter’
‘There are many attractive natural sights in my neighborhood (such as (front) gardens, landscaping, views)’
There are attractive buildings/homes in my neighborhood

*Traffic safety*

‘There is a lot of traffic along the street I live in’
‘The speed of traffic on the street where I live is usually fast’
‘Most drivers exceed the posted speed limits while driving in my street’
‘I like to walk along the street where I live’
‘On this street, I feel safe from crime’
‘On this street, I feel safe from road accidents’
‘Crossing the road is safe for pedestrians’
